# Targeting CD4⁺ T cell infiltration: mechanisms and therapeutic insights into CD4⁺ T cell transmigration across the blood–brain barrier in acute ischemic stroke

**DOI:** 10.1186/s12987-026-00821-6

**Published:** 2026-06-17

**Authors:** Congai Chen, Jialin Cheng, Zehan Zhang, Jun Zhou, Shuling Liu, Ying Liu, Chuxin Zhang, Yuxiao Zheng, Yiping Wu, Qinyuan Zhang, Lu Liu, Changxiang Li, Bin Li

**Affiliations:** 1 Beijing Hospital of Traditional Chinese Medicine, Beijing, 100010 China; 2 Beijing University of Chinese Medicine, Beijing, 102488 China; 3 Dongzhimen Hospital Beijing University of Chinese Medieine, Beijing, 100700 China

**Keywords:** Ischemic stroke, CD4⁺ T cell, Blood–brain barrier (BBB), Transendothelial migration (TEM), Neuroinflammation

## Abstract

The blood-brain barrier (BBB) serves as a physical barrier of the central nervous system, characterized by selective permeability and immune isolation. It plays a critical role in transporting essential nutrients for normal neuronal metabolism while preventing the entry of toxic substances into the brain. Following acute ischemic stroke, the integrity of the BBB is compromised, leading to extensive T-cell infiltration into the brain. These infiltrating T cells influence BBB permeability and can exert either protective or detrimental effects on brain tissue. Current research has largely focused on the subset-specific regulation of T cells after their infiltration or on inhibiting T-cell migration into the brain during ischemic stroke. However, a systematic understanding of the routes and mechanisms underlying T-cell trafficking across the BBB in this context remains limited. In this review, we systematically examine the functional alterations of the BBB induced by different CD4^+^ T-cell subsets in ischemic stroke and explore the associated molecular mechanisms. Special emphasis is placed on the migratory behavior of CD4^+^ T cells across the BBB, highlighting the roles of selectins, LFA-1, ICAM, and VCAM in mediating T-cell capture, adhesion, and transmigration. We also provide a detailed discussion of immunosuppressive agents clinically used to inhibit the infiltration of peripheral immune cells into the brain. By synthesizing these insights, this review aims to inform the optimization of therapeutic strategies targeting CD4^+^ T-cell trafficking and to facilitate the development of adjunct neuroprotective approaches, thereby advancing stroke treatment and improving patient outcomes. Ultimately, this work seeks to deepen the understanding of the mechanisms by which CD4^+^ T cells influence ischemic stroke and to provide a theoretical foundation for novel immunomodulatory neuroprotective therapies.

## Introduction

Stroke has emerged as a major global public health challenge due to its high incidence, disability rate, and mortality. Ischemic stroke (IS) accounts for approximately 70% of all stroke cases [1]. Blood-brain barrier (BBB) disruption is a key pathological factor contributing to poor outcomes in patients with acute ischemic stroke (AIS). Although modern medicine has made advances in the treatment of AIS, including revascularization therapies that effectively restore cerebral blood flow [2], these treatments may exacerbate BBB damage and increase the risk of hemorrhagic transformation [3]. Despite extensive research focused on BBB repair, no strategy has yet achieved recognized clinical efficacy. A major reason for this bottleneck is that therapies targeting a single pathway are inadequate to counteract the complex, multi-mechanistic injury cascades triggered following IS [4].

In recent years, a growing body of research has suggested that therapies targeting peripheral immune cells hold promise as potential strategies for improving the management of IS [5]. In the healthy human brain, where the BBB remains intact, only a limited number of T cells enter the central nervous system (CNS). These cells collaborate with resident immune cells to conduct immune surveillance and help maintain CNS homeostasis [6]. However, following AIS, disruption of the BBB allows uncontrolled infiltration of blood-borne cells, macromolecules, and fluid into the brain. This breach facilitates the rapid migration of large numbers of peripheral immune cells to the ischemic area, where they participate in local immune responses [7].

T lymphocytes serve as principal cellular components of the adaptive immune system and play a central role in mediating immune responses [8]. Among them, CD4⁺ T cells represent the predominant effector subset [9] and exert a pivotal influence throughout the pathological progression of IS [10]. Clinical observations indicate that peripheral T lymphocyte counts in patients with AIS decrease exponentially within seven days after onset, reaching a nadir as early as 12 h [11]. This decline coincides with substantial T-cell infiltration into the damaged cerebral hemisphere, where these cells accumulate extensively. CD4⁺ T cells begin to infiltrate the brain approximately 24 h post-ischemia, peak around 72 h [12], and can persist for up to one month [13], highlighting their crucial role in determining disease prognosis. Accumulating evidence underscores the essential, yet dualistic functions of T lymphocytes in the initiation and evolution of cerebral infarction. In the early phase following stroke, infiltrating T cells promote inflammatory injury within the affected brain tissue. Experimental studies demonstrate that mice deficient in CD4⁺ helper T cells or CD8⁺ T cells develop significantly smaller infarct volumes [14]. Moreover, selective antibody-mediated depletion of either CD4⁺ or CD8⁺ T cells markedly reduce infarct size in permanent middle cerebral artery occlusion (MCAO) models [15], confirming their detrimental role during acute injury. Conversely, during later stages of infarction, certain T-cell subsets contribute to tissue repair and functional recovery [16]. Notably, regulatory T cells (Tregs), a specialized subpopulation of CD4⁺ T helper cells, mediate neuroprotection and improve neurological outcomes through the secretion of anti-inflammatory cytokines. Recent research further reveals that brain-homing Tregs recruited during the chronic phase of stroke suppress the formation of neurotoxic astrocytes and attenuate astrogliosis, thereby supporting functional restoration [17]. Collectively, these findings suggest that therapeutic strategies selectively targeting specific T-cell subpopulations may offer promising avenues for improving post-stroke outcomes.

Given that the migration of T cells into the CNS is tightly regulated by the BBB mechanism, this process remains highly controlled under physiological conditions. However, during neuroinflammatory states, immune cells can overcome this barrier and infiltrate the parenchyma of the CNS. The transmigration of peripheral immune cells into the brain is a complex multi-step process, involving a diverse array of adhesion molecules and immune mediators [18]. To advance our understanding of the role of CD4⁺ T cells in IS, this review systematically summarizes the functional characteristics of different CD4⁺ T cell subsets and elucidates their specific mechanisms in mediating BBB dysfunction and infiltrating the CNS following cerebral ischemia. Furthermore, we provide a detailed overview of immunomodulatory agents that target T cells or their trafficking processes. By integrating these insights, this review aims to enhance the mechanistic understanding of T cell involvement in IS and facilitate the development of novel therapeutics directed against these immune players.

## An overview of CD4^+^ T lymphocytes

**Fig. 1:**
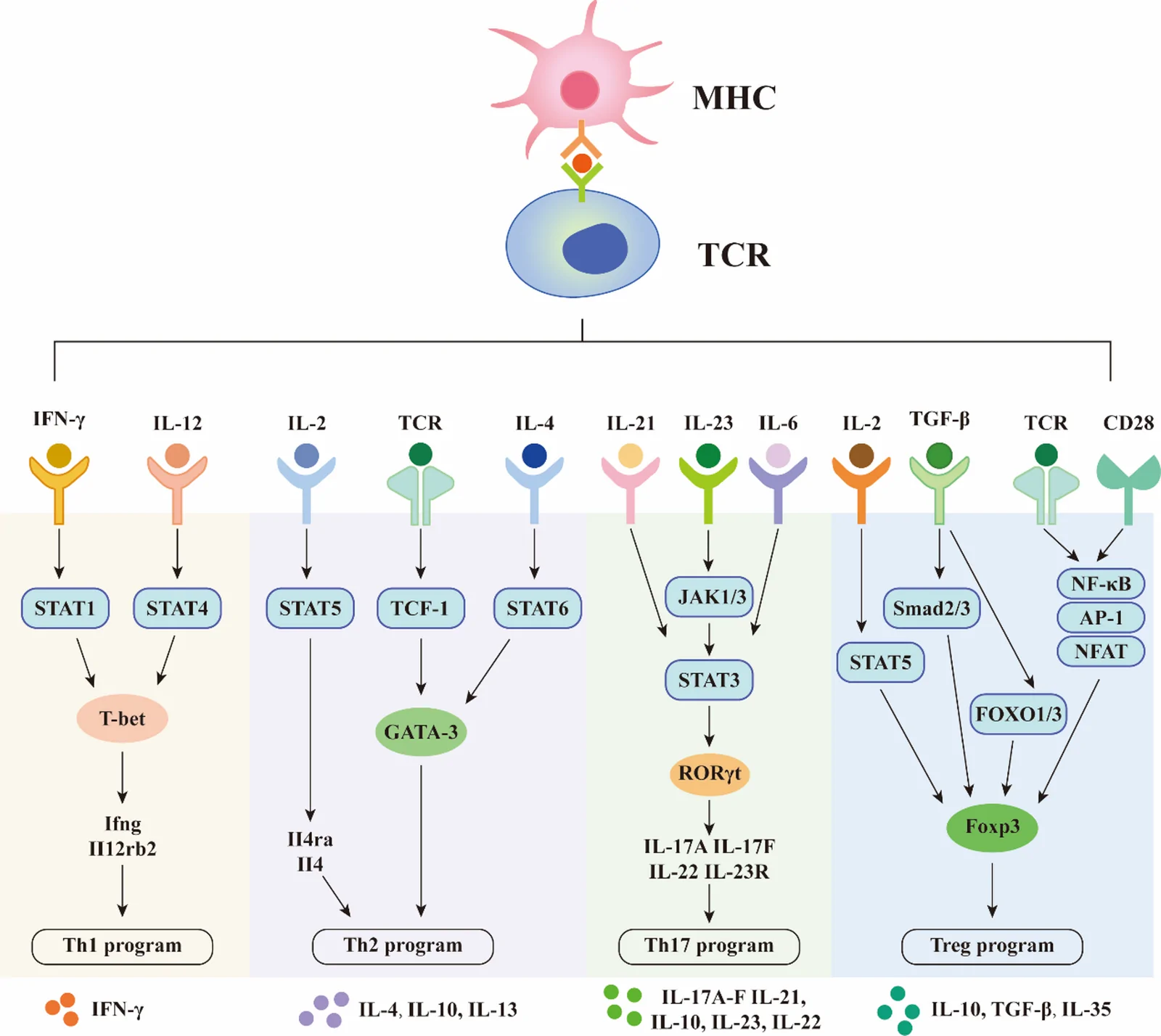
The main phenotypes of CD4^+^ T lymphocytes and the stimulating factors. CD4^+^ T lymphocytes undergo a series of phenotypic changes during their development, activation, and memory formation. Their differentiation is initiated when the T cell receptor (TCR) engages with an antigenic peptide-MHC complex, providing the primary activating signal. This initial event is followed by co-stimulation, downstream signaling networks, and the cytokine microenvironment, which collectively orchestrate their differentiation into distinct lineages such as Th1, Th2, Th17, and Treg cells

To defend against innumerable pathogenic invasions, mammals have evolved a sophisticated and precisely regulated immune system, composed of diverse cellular components, cytokines, chemokines, antibodies, and other molecular mediators [19]. As a central component of this system, CD4^+^ T lymphocytes play a pivotal role throughout life by orchestrating the actions of other immune and non-immune cells, thereby influencing nearly all aspects of host defense and immune regulation [20]. CD4^+^ T cells originate from common lymphoid progenitors in the bone marrow and undergo development in the thymus, with their primary functions being executed in peripheral tissues and lymphoid organs [21]. This developmental process is tightly regulated, enabling CD4^+^ T cells to effectively recognize a broad spectrum of foreign antigens while avoiding auto-reactivity against self-tissues. To perform their effector functions, naïve CD4^+^ T cells must first be activated upon receiving pathogen signals from antigen-presenting cells (APCs), such as dendritic cells. Following activation, they differentiate into distinct T effector subsets according to the nature of the immune challenge [22]. Based on their characteristic cytokine profiles and lineage-defining transcription factors, CD4^+^ T helper cells are conventionally classified into four major subsets: T helper 1 (Th1, producing IFN-γ and expressing T-bet), T helper 2 (Th2, producing IL-4, IL-5, IL-13 and expressing GATA3), T helper 17 (Th17, producing IL-17, IL-22 and expressing RORγt), and Tregs (producing IL-10, TGF-β, IL-35 and expressing Foxp3) [23](Fig. 1). Additional subsets have also been described in studies, such as Th3 (TGF-β) [24], Tr1 (IL-10) [25], Th9 (IL-9) [26, 27], and Th22 (IL-22) [28]. Among these, Th1, Th2, Th17, and Treg cells have been widely implicated in the pathogenesis and progression of IS.

### Th1 cells

Under specific cytokine conditions, naïve CD4⁺ T cells can differentiate into the Th1 cells following T-cell receptor activation. This polarization is primarily driven by interleukin-12 (IL-12) secreted by APCs, which activates the transcription factor STAT4. Additionally, IFN-γ, which is produced by NK cells or T cells themselves, activates STAT1. Both STAT1 and STAT4 signaling converge to induce the expression of T-bet, the master transcription factor defining Th1 lineage commitment [29]. Upon maturation, Th1 cells secrete pro-inflammatory cytokines such as IFN-γ, IL-2 and various chemokines. Among them, IL-2 can upregulate the expression of the Th1 lineage-specific transcription factor T-bet in CD4^+^ T lymphocytes, and it can produce a synergistic effect with IFN-γ, thereby forming a positive feedback loop that promotes the continuous release of Th1-type cytokines [30]. In addition, the large amount of secreted IFN-γ will promote the production of reactive oxygen species and nitric oxide, which contribute to BBB disruption [31].

### Th2 cells

Naïve CD4⁺ T cells can differentiate into the Th2 cells upon activation by signals from dendritic cells [32]. Although IL-2 broadly supports the expansion of T effector subsets, it plays an additional role in Th2 polarization by activating STAT5; concurrently, IL-4 activates STAT6, which serves as the lineage-defining signal for Th2 commitment [33]. STAT6 in turn induces the expression of GATA3, the master transcription factor for Th2 cell fate. GATA3 not only drives Th2 differentiation but also suppresses the expression of key transcription factors associated with alternative T-cell lineages, such as T-bet (Th1) and RORγt (Th17) [19]. Mature Th2 cells secrete cytokines such as IL-4, IL-5, IL-10, and IL-13, which are essential for orchestrating effective inflammatory immune responses against extracellular pathogens (e.g. parasites), most notably by promoting B cell class-switching to IgE production and the activation of mast cells and eosinophils. Importantly, in the CNS these cytokines also exhibit notable anti-inflammatory properties, including shifting the response patterns of macrophages and microglia toward reparative phenotypes. In the context of cerebral ischemia, they contribute to multiple protective processes, including the production of nerve growth factor, clearance of cellular debris, tissue remodeling and repair, and the promotion of angiogenesis [34].

### Th17 cells

Th17 cell differentiation is a multi-stage process precisely orchestrated by specific cytokine milieus and the master transcription factor RORγt, leading to the production of characteristic cytokines including IL-17 A, IL-17 F, IL-22, and GM-CSF [35]. The process initiates when TGF-β and IL-6 (or alternatively IL-21 [36]) from APCs drive naïve T cells toward the Th17 lineage. This is followed by an amplification phase where autocrine IL-21 creates a positive feedback loop, promoting clonal expansion and upregulation of the IL-23 receptor [37]. Terminal maturation occurs upon IL-23 signaling, which stabilizes the pathogenic phenotype and enhances production of effector cytokines like IL-17 A [38]. Functionally, IL-17 A disrupts blood-brain barrier integrity by targeting endothelial tight junctions, facilitating extensive CD4^+^ T cell infiltration into the CNS parenchyma [35]. Moreover, IL-17 A can induce excessive neuronal autophagy, thereby aggravating ischemic injury in transient MCAO models [39]. Notably, Th17 cells exhibit considerable phenotypic plasticity in response to microenvironmental cues. Under pro-inflammatory conditions, particularly in the presence of IL-23, Th17 cells (IL-17 A^+^IFNγ^−^) can progressively transition through an intermediate IL-17 A^+^IFNγ^+^ co-producing state and ultimately give rise to ex-Th17 cells (IL-17 A^−^IFNγ^+^) that phenotypically resemble Th1 cells [40, 41]. Furthermore, after IL-1β binds to the IL-1R1 on the surface of T cells, some Th17 cells can simultaneously express IL-17 A and IFN-γ, thereby exhibiting the dual characteristics of Th1 and Th17 cells [42]. This indicates that Th17 cells are not in a terminally differentiated state, but rather have high plasticity in the inflammatory microenvironment. Their functional profile can dynamically change in response to local immune signals.

### Treg cells

Treg cells, identified as CD4⁺ CD25⁺ and defined by the expression of the transcription factor Foxp3, originate from CD4⁺ T cell precursors. Their differentiation is primarily induced by TGF-β and IL-2. Under physiological conditions, Tregs are generated in the thymus but can also differentiate from conventional naïve T cells in peripheral lymphoid organs. In the context of IS, the population of Foxp3⁺ Tregs in the thymus increases one week after onset, coinciding with their observed infiltration into the ischemic hemisphere [43]. Tregs are characterized by high expression of the CD25 and the production of anti-inflammatory factors such as IL-10, TGF-β, and IL-35, alongside the lineage-defining transcription factor Foxp3 [44]. Accumulating evidence indicates that Tregs contribute to post-stroke repair and suppress neuroinflammation [45]. Studies have demonstrated that CD4⁺ Tregs play a beneficial role by limiting excessive inflammation, attenuating blood-brain barrier disruption, protecting neurons, and promoting neurogenesis during the acute and subacute phases of IS. During the recovery phase, they further facilitate neural repair processes [43].

## CD4^+^ T lymphocyte infiltration and their role in modulating BBB integrity following IS

Following the onset of AIS, innate immune cells are activated first, after which T cells become activated and infiltrate the brain tissue. The process of T cell infiltration into the ischemic brain begins within 24 h post-stroke and can persist for up to four weeks [46]. The most pronounced phase of lymphocyte infiltration occurs between days 3 and 7 after the ischemic event [47]. Different T cell subsets infiltrate the brain at distinct time points, a process regulated by the specific actions of various cytokines and chemokines [48]. CD4^+^ T cells infiltrate the infarcted area as early as day 1, increase substantially thereafter, and peak around day 7. Certain CD4^+^ T cell subtypes continue to enter the infarct region even 7 days after AIS [49]. Among the various CD4^+^ T helper subsets, Th1 and Th17 cell numbers increase during the first 3 days and decline by day 7, whereas Th2 cells exhibit an opposite temporal pattern [50].

Following ischemic stroke, T cells primarily infiltrate the brain parenchyma through three routes: the BBB, choroid plexus, and meninges [48]. Among these, the BBB, which is formed by microvascular endothelial cells of cerebral capillaries, constitutes the largest interface between the circulation and the brain [51, 52]. During the early phase of acute ischemic stroke, CD4^+^ T cells accumulate near endothelial cells and release cytokines including IL-1β, TNF-α, IL-6, IL-10, IFN-β, TGF-β, and MMP-9. These inflammatory mediators contribute to tight junction degradation and compromise BBB integrity [53]. Specifically, Th1 cells promote tissue damage through the production of pro-inflammatory cytokines such as IFN-γ, which enhance immune cell infiltration and activate inflammatory responses [54]. Th17 cells produce IL-17, which impairs the BBB by downregulating occludin and ZO-1 expression while promoting the recruitment of monocytes and neutrophils through upregulation of CCL2 and CXCL1 in endothelial cells [55]. In contrast, anti-inflammatory T cells such as Tregs and Th2 cells protect the ischemic hemisphere by suppressing excessive inflammation in the early phase and facilitating neural repair in later stages [23, 56]. Tregs primarily upregulate IL-10 and TGF-β to inhibit the hyperactivation of resident microglia and infiltrating T cells, thereby reducing levels of pro-inflammatory factors including TNF-α, IFN-γ, and IL-1β [57]. Through programmed death-ligand 1-mediated transcellular interaction with neutrophils, Tregs directly suppress MMP-9 production, contributing to preserved BBB integrity and improved neurological function [58]. Furthermore, Tregs significantly attenuate BBB damage and functional impairment by downregulating endothelial CCL2 and MMP-9 expression [59].

## Impact of CD4^+^ T lymphocyte infiltration on the blood-brain barrier following AIS

### Detrimental and beneficial effects of CD4^+^ T cell subsets on the BBB (Th1, Th17, Treg, Th2)

#### Th1:

CD4^+^ T cell-mediated brain injury following stroke is primarily driven by the pro-inflammatory effects of Th1 cells. The differentiation of this CD4^+^ subset is initiated by IL-12 and IFN-γ under the specific regulation of the transcription factor T-bet [60]. Th1 cells contribute to tissue damage mainly through the production of multiple pro-inflammatory cytokines, including IL-2, IL-21, TNF-α, and IFN-γ, which collectively promote immune cell infiltration and activate inflammatory responses. IL-2 secreted by Th1 cells enhances the activation of T cells, B cells, and macrophages, and induces cytotoxicity in CD8^+^ T cells [60]. IL-21 interacts with neuronal IL-21 receptors, upregulating the mRNA expression of ATG6 and inducing autophagic processes that exacerbate tissue injury [61]. TNF-α participates in various inflammatory pathways, activating and recruiting neutrophils. It also promotes the shift of microglia toward a pro-inflammatory phenotype, leading to the release of inflammatory mediators such as IL-1, IL-6, IL-12, and nitric oxide, thereby increasing vascular permeability [54]. IFN-γ disrupts tight junction integrity at the blood-brain barrier and facilitates Th1 infiltration through the activation of IFN-γ-induced proteins [62]. Additionally, IFN-γ activates macrophages and amplifies the secretion of cytokines including TNF-α [63]. In atherosclerotic vessels, Th1-derived TNF-α and IFN-γ act on vascular smooth muscle cells, suppressing collagen synthesis and promoting plaque instability, which increases the risk of intraplaque hemorrhage [64]. Th1 cells drive the polarization of microglia toward a pro-inflammatory phenotype via TNF-α and IFN-γ [65]. These pro-inflammatory microglia secrete IL-12 and TNF-α, and express chemokines such as CXCL9 and CXCL10, which in turn promote the recruitment and activation of Th1 cells [66], thereby establishing a self-amplifying inflammatory loop.

#### Th17: 

Th17 cells represent another effector CD4^+^ T cell subset characterized by their production of the pro-inflammatory cytokines IL-17 and IL-21. However, during the early phase of stroke, IL-17 is primarily secreted by γδ T cells [67] and contributes to blood-brain barrier disruption by upregulating the expression of MMP-3 and MMP-9 in endothelial cells [68]. Recent clinical evidence indicates that elevated peripheral IL-17 levels within three days after stroke onset correlate with worsened neurological outcomes [69]. Neutralizing IL-17 has been shown to partially reduce the infiltration of neutrophils and CD8^+^ T cells, as well as attenuate γδ T cell-induced BBB damage [63, 68]. Through IL-17 secretion, Th17 cells collaborate with lymphotoxin α1β2 and various CCL and CXCL chemokines to orchestrate the clustering of myeloid cells around T and B cells, facilitating the formation of ectopic lymphoid structures that promote B cell expansion and differentiation [10]. Th17 cells also contribute to brain injury *via* IL-17-mediated crosstalk with M1-polarized microglia [70]. Interestingly, Th17 cells produce IL-26, a cytokine demonstrated to protect BBB integrity in in vitro models by upregulating the expression of occludin, claudin-5, and ZO-1 [71]. Nevertheless, whether IL-17 itself exerts protective effects on the intact BBB during cerebral infarction remains unclear and requires further investigation.

#### Treg: 

During the early phase following cerebral ischemia, circulating Treg levels decrease significantly in peripheral blood, while Tregs progressively accumulate within the ischemic region of the central nervous system. This migration initiates with Treg adhesion to cerebral vessels between days 1 and 4, followed by parenchymal infiltration starting around day 5 [72]. This process continues until day 28 [73], with elevated Treg levels maintained for up to two months in the brain [17]. Regulatory T cells exhibit dual, time-dependent functions in AIS. During the very early hyperacute phase, Tregs accumulating in the cerebral microvasculature within hours after cerebral blood flow cessation can paradoxically promote microvascular dysfunction. During the early phase of AIS, reciprocal adhesion between T cells and platelets within the microvasculature may contribute to microvascular dysfunction and thrombus formation. Platelet-derived CD84 accumulates in the microvessels and binds to CD84 on CD4^+^ T cells, thereby promoting T cell recruitment [74]. Tregs accumulate in the cerebral microvasculature within hours after cerebral blood flow cessation. Through the CD40/CD40L pathway, they interact with both endothelial cells and platelets, inducing thrombus formation and promoting stroke progression, a process known as thromboinflammation [75]. In 2013, Kleinschnitz et al. first reported that within 24 h after transient MCAO, Tregs interact with endothelial cells in the ischemic brain via the LFA-1/ICAM-1 signaling pathway, leading to microvascular dysfunction. This detrimental effect was shown to be platelet-dependent, as Treg-platelet interactions resulted in reduced cerebral blood flow, increased fibrinogen deposition in brain tissue, and ultimately larger infarct volumes and enhanced thrombosis [76]. Subsequently, Schuhmann et al. confirmed these findings, demonstrating that increased Treg numbers exacerbate thromboinflammation in a transient stroke model, thereby worsening stroke outcomes [75]. Beyond this hyperacute, intravascular detrimental window, however, accumulating evidence underscores the predominantly protective role of Tregs in AIS [77], a beneficial effect that is well documented and strongly supported by numerous studies in permanent ischemia models. The protective functions of Tregs involve immunosuppression, blood-brain barrier preservation, and neural repair [78]. They may improve stroke outcomes by modulating gut microbiota to suppress IL-17^+^ γδ T cell proliferation [79], and their suppression of immune activation helps mitigate the detrimental impact of inflammatory responses on BBB integrity [80]. Through inhibition of neutrophils and inflammatory microglia, Tregs significantly reduce BBB disruption by attenuating MMP-9 and reactive oxygen species production. They also downregulate endothelial CCL2 expression, limiting the retention of other immune cells within the ischemic area and thereby protecting the BBB [59]. Consequently, adoptive transfer of Tregs to directly increase their numbers has emerged as an effective therapeutic strategy for T cell–related stroke treatment [59].

#### Th2: 

Under the specific regulation of the transcription factor GATA-3, CD4^+^ T cells differentiate into the Th2 subset [60]. Th2 cells primarily contribute to immunosuppressive and reparative activities after stroke through the production of cytokines such as IL-4, IL-10, IL-35, and TGF-β [81]. The anti-inflammatory factors secreted by Th2 cells significantly inhibit the functions of Th1 and Th17 cells. Furthermore, key Th2-derived cytokines, including IL-4, IL-5, and IL-13, help prevent atherosclerosis and may reduce the risk of intraplaque rupture, thereby lowering the incidence of hemorrhagic transformation [64]. These anti-inflammatory cytokines also promote the polarization of microglia toward the M2 phenotype [65, 82, 83], increasing their production of insulin-like growth factor and neurotrophic factors, which enhances microglia-mediated neuroprotection [84, 85]. In a reciprocal manner, M2-polarized microglia can secrete IL-4, CCL17, CCL22, and CCL24, which in turn promote the recruitment and activation of Th2 cells [65].

### Temporal dynamics of CD4⁺ T cell effects on the blood-brain barrier following ischemic stroke

The impact of CD4⁺ T cells on blood-brain barrier integrity evolves in a temporally structured manner following ischemic stroke, with distinct subsets exerting opposing effects on BBB permeability, tight junction integrity, and endothelial function at different post-stroke phases.

Acute phase: During the acute phase, the BBB sustains its most severe structural damage, with permeability peaking at 6–48 h after AIS onset and correlating with maximal edema formation and hemorrhagic transformation risk [86]. CD4⁺ T cells begin infiltrating the brain within 24 h, with Th1 and Th17 cells as the predominant subsets [87]. Th1 cells exacerbate BBB breakdown through IFN-γ-mediated suppression of claudin-5 and ZO-1, and upregulation of endothelial ICAM-1 and VCAM-1 [62]. Th17 cells compound this injury via IL-17 A-driven downregulation of occludin and ZO-1, and stimulation of MMP-3 and MMP-9 secretion, progressively degrading the basement membrane and widening the paracellular route for immune cell entry [68]. Although Treg cells are rarely detected within the parenchyma at this stage, CCR5⁺ Tregs in peripheral circulation dock onto CCL5-expressing ischemic endothelium from the luminal side, suppressing neutrophil-derived MMP-9 via PD-L1 signaling and downregulating endothelial CCL2, thereby providing early BBB protection without parenchymal entry [58, 88].

Subacute phase: The cytokine microenvironment shifts as IL-6 and IL-33 promote Th2 and Treg differentiation while suppressing Th1 and Th17 activity, reducing the proportion of subsets that drive tight junction degradation [87]. Treg cells begin parenchymal infiltration around day 5, increasing significantly from day 7 [72]. In the subacute stage, Tregs inhibit neutrophil-derived MMP-9 via the PD-1/PD-L1 pathway and downregulate endothelial CCL2, progressively alleviating BBB disruption and limiting further pro-inflammatory immune cell retention [59, 87].

Chronic phase: Elevated Treg levels are maintained for up to two months in the ischemic brain [17]. Treg cell-derived osteopontin acts through integrin receptors on microglia to enhance microglial reparative activity, consequently promoting oligodendrogenesis and white matter repair. Increasing the number of Treg cells can improve the white matter integrity after stroke and restore neurological function in the long term [89]. Th2 cells contribute to this restorative phase through IL-4-driven M2 microglial polarization, reducing endothelial-damaging cytokines and supporting tight junction re-expression [78]. Therapeutic strategies targeting T cell infiltration must therefore be designed with phase specificity in mind.

### Influence of sex and aging on CD4⁺ T cell responses in ischemic stroke

The functional profile of CD4⁺ T cells in ischemic stroke is fundamentally shaped also by two clinically critical biological variables, sex and aging, that modulate both baseline immune composition and the neuroimmune response to injury.

#### Sex differences in CD4⁺ T cell responses after stroke

Sex is a fundamental biological variable that shapes CD4⁺ T cell responses to ischemic injury and their downstream effects on blood-brain barrier integrity. Estrogen exerts opposing effects through two receptor subtypes: ERα signaling promotes Th17 differentiation by enhancing IL-17 A production and upregulating IL-23 receptor expression, while ERβ signaling suppresses Th17 differentiation and promotes Treg induction [90]. In contrast, androgens attenuate Th1 responses in CD4⁺ T cells by reducing IFN-γ production [91]. A critical caveat concerns menopausal status: the protective effects of estrogen on CD4⁺ T cell subset balance, particularly ERβ-driven Treg expansion and Th17 suppression, are predominantly operative during the premenopausal phase [92]. Following menopause, the loss of estrogen-mediated immunomodulation may permit greater Th17/Treg imbalance, which is consistent with the clinical observation of worse post-stroke functional outcomes in older women despite the theoretical immunological advantages conferred by female sex [92]. These findings collectively underscore the importance of sex- and hormonal status-stratified approaches in clinical trials targeting CD4⁺ T cell-BBB interactions after stroke.

#### Aging and immunosenescence: impact on CD4⁺ T cell responses in stroke

Stroke affects the elderly, yet most preclinical immunological studies employ young animals, creating a critical translational gap. Aging remodels the CD4⁺ T cell compartment through immunosenescence, characterized by thymic involution, naïve T cell pool contraction, and memory subset expansion, shifting the balance toward Th1 and Th17 dominance and elevating systemic inflammatory tone [93]. At the molecular level, autophagy defects in aged CD4⁺ T cells increase mitochondrial ROS production and promote Th17 differentiation via STAT3 activation, while elevated basal NF-κB activity drives constitutive pro-inflammatory cytokine transcription [94]. The Treg/Th17 balance, essential for limiting post-stroke neuroinflammation, is progressively disrupted with aging: the Th17/Treg ratio increases in elderly individuals, amplifying the pro-inflammatory baseline upon which ischemic injury is superimposed [95]. Although Treg numbers may paradoxically increase with aging, their suppressive capacity is markedly impaired compared to those of younger counterparts [96]. Future preclinical studies must systematically incorporate aged and sex-matched models, and clinical trials should include rigorous age- and sex-stratified analyses to ensure that immunomodulatory strategies are applicable to the high-risk elderly population most affected by ischemic stroke.

## The process of CD4^+^ T lymphocyte trafficking across the blood-brain barrier

**Fig. 2:**
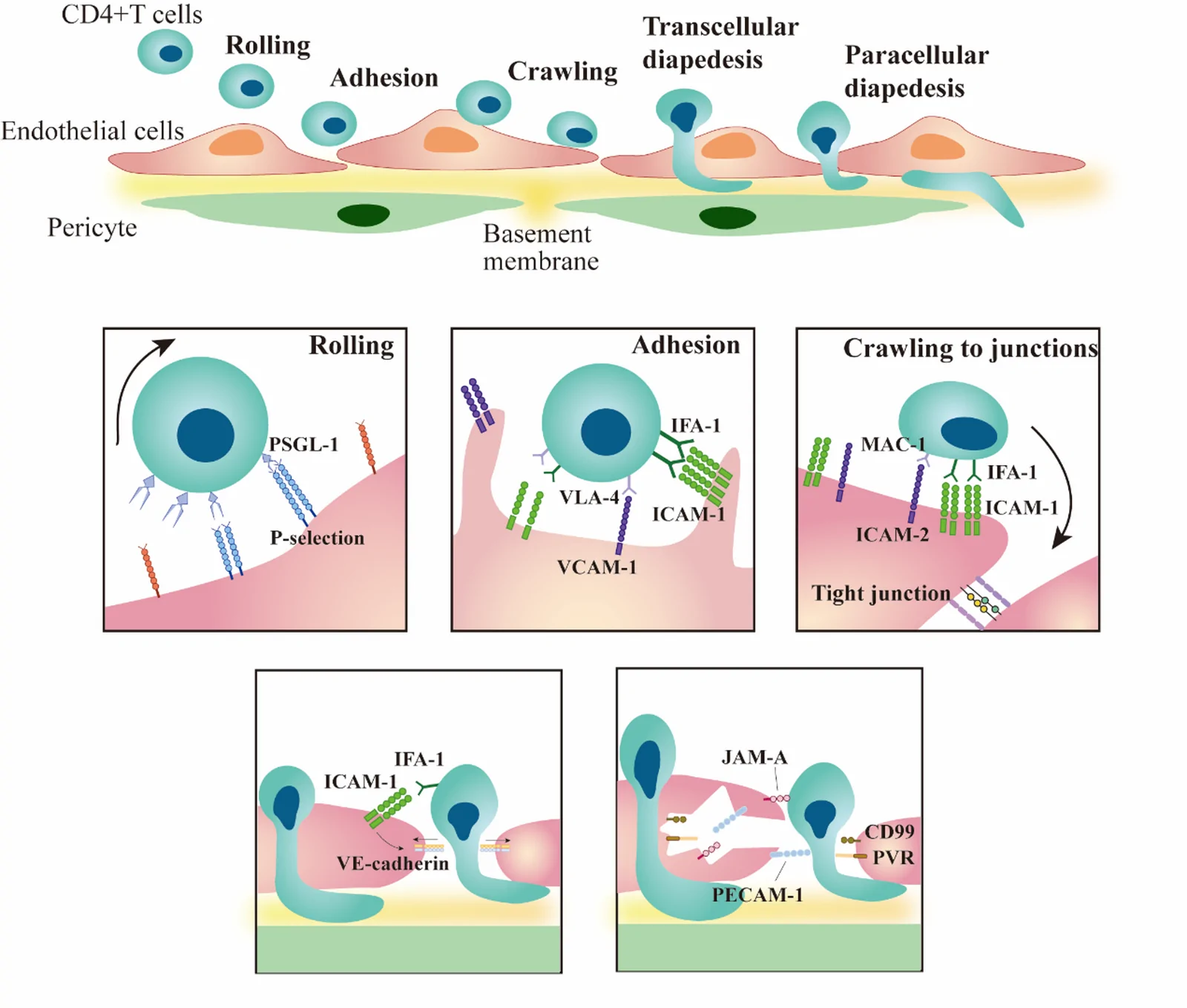
The process of CD4^+^ T cells infiltrating the blood-brain barrier. The traversal of T cells across the blood-brain barrier is an orchestrated multi-step cascade. It commences with selectin-mediated tethering and rolling on the cerebral endothelium, progresses to integrin-dependent firm adhesion and crawling (e.g., via LFA-1/ICAM-1 or VLA-4/VCAM-1 interactions), and culminates in diapedesis through either paracellular or transcellular mechanisms

Following the recognition of the critical role played by CD4^+^ T cell infiltration into the central nervous system, there is a need to identify potential therapeutic targets for intervention. Achieving this first requires a comprehensive understanding of the process and specific mechanisms by which CD4^+^ T cells traverse the BBB. Under homeostatic conditions, T cells circulate rapidly through blood vessels. However, during cerebral infarction, the interaction between T cells and inflamed cerebral endothelium follows a sequential three-step process: initial capture and rolling, followed by firm adhesion and crawling, and finally transmigration across the endothelial barrier [97] (Fig. 2). This directed migration of T cells into the CNS parenchyma marks the initiation of the adaptive immune response in cerebral infarction.

### Capture and rolling

For leukocytes to initiate transendothelial migration (TEM), they must first pass a critical checkpoint: establishing contact with activated endothelial cells under inflammatory conditions. Endothelial selectins play a fundamental role in this process. When T cells recognize antigens and initiate autoimmune inflammatory events, the binding of selectins on cerebral microvascular endothelial cells to their T cell ligands mediates the initial capture and subsequent rolling of T cells along the vascular lumen [98]. Selectins comprise a family of three closely related cell adhesion molecules: P-selectin expressed on platelets and endothelial cells; E-selectin expressed on vascular endothelium; and L-selectin expressed on leukocytes [99]. Following cerebral infarction, endothelial P-selectin and E-selectin are significantly upregulated in response to cytokines released by innate immune cells [100]. The primary ligand for P-selectin, P-selectin glycoprotein ligand-1 (PSGL-1), is constitutively expressed across all T-cell subsets [101]. PSGL-1 serves as a high-affinity ligand for selectin family members, primarily P-selectin and E-selectin, enabling rapid leukocyte adhesion [102]. Thus, when E-, P-, and L-selectins engage PSGL-1, T cells become captured and roll along the endothelial surface at reduced velocities [103]. Another P-selectin ligand, T-cell immunoglobulin and mucin domain 1, also functions as a major selectin ligand that regulates leukocyte rolling on P-selectin in vivo. T-cell immunoglobulin and mucin domain 1 cooperates with PSGL-1 to mediate the capture and rolling of Th1 and Th17 cells within inflamed cerebral microvessels [104]. E-selectin ligands expressed on T cells, including CD43 and CD44, contribute to Th1 cell adhesion under inflammatory conditions [105]. This phase, commonly referred to as the rolling phase, is essential for enabling T cells to progress to the subsequent step of TEM, namely firm adhesion. Evidence supporting the physiological relevance of this process includes studies demonstrating that elevated levels of soluble P-selectin in mice can disrupt BBB integrity and exacerbate stroke severity in the context of atherosclerosis [106].

### Adhesion and crawling

Capture and rolling on the endothelial surface facilitate interactions between chemokines and their corresponding receptors. These signaling molecules are specifically presented on the surface of inflamed endothelial cells and induce inside-out signaling that activates integrins to their high-affinity state, ultimately leading to T cell arrest. Among the integrins involved, the most extensively studied are lymphocyte function-associated antigen 1 (LFA-1; αLβ2) and very late antigen 4 (VLA-4; α4β1). Under physiological conditions, endothelial cells express low levels of intercellular Adhesion Molecule − 1(ICAM-1), ICAM-2, and VCAM-1; however, their expression is markedly upregulated following cerebral infarction [107], enabling interactions with T cell integrins that regulate CD4^+^ T cell migration across the blood-brain barrier [108]. T cell adhesion mediated by integrin-ligand binding, such as LFA-1/ICAM-1 or VLA-4/VCAM-1, has been demonstrated in multiple in vivo and in vitro studies [109–111]. However, many of these investigations were conducted under static conditions, overlooking the impact of physiological shear flow. Recent advances using live imaging techniques have revealed that within 3 min of arrest on cytokine-stimulated primary brain endothelial cells, encephalitogenic Th1 cells polarize and begin crawling against the direction of flow, actively seeking permissive sites for transmigration [112]. This arrest and crawling behavior on cerebral endothelium involves both VLA-4/VCAM-1 and LFA-1/ICAM-1 interactions, with Th1 and Th17 cell polarization and crawling specifically mediated by LFA-1 binding to endothelial ICAM-1 and ICAM-2 [112, 113]. Notably, when VLA-4 is blocked, lymphocytes can utilize melanoma cell adhesion molecule (MCAM) as an alternative pathway for CNS migration [114]. In the blood-brain barrier, MCAM expression is predominantly induced during autoimmune neuroinflammation and preferentially enhances rolling, adhesion, and migration of pathogenic lymphocytes (Th1 and Th17 cells) into the CNS. ST14, a ligand for MCAM, has been shown to regulate CD4^+^ T cell infiltration across the BBB [115]. Additionally, the chemokines CCL19 and CCL21 in the blood enhance T cell adhesion and crawling on endothelial cells by engaging CCR7 expressed on immune cells [116]. Following adhesion, T cells typically undergo extensive crawling to locate suitable sites for transmigration. This process represents a distinctive feature of the highly specialized CNS vascular barrier, which tightly regulates leukocyte entry [117].

### Transendothelial migration and parenchymal penetration

Although endothelial ICAM-1 and ICAM-2 are essential for T cell crawling, a small number of T cells can still traverse cerebral endothelial monolayers even in the absence of these adhesion molecules [112]. This observation indicates that additional pathways facilitate T cell penetration across the endothelial barrier following initial adhesion and crawling [118]. After T cell adhesion, ligand binding to integrins in their high-affinity state triggers outside-in signaling, leading to cytoskeletal reorganization that strengthens cellular adhesion (termed firm adhesion) and induces T cell spreading characterized by expansion and uropod formation [119, 120]. Following adhesion, integrins further guide T cells to migrate through endothelial cells *via* the transcellular route or between them *via* the paracellular route to reach underlying tissues. T cells form specialized podosomes, which are protrusive structures characterized by an outer ring containing LFA-1 and talin-1 and an inner core rich in F-actin. The LFA-1 within these podosomes interacts with ICAM-1-enriched invaginations (podoprints) on the endothelial surface, forming transcellular pores that facilitate T cell transmigration [121]. Additionally, LFA-1 binds to apically located junctional adhesion molecule-A and tight junctions of endothelial cells, thereby directing the paracellular penetration of T cells [122]. Experimental evidence confirms that Th17 cell infiltration into the brain parenchyma, which promotes direct neuronal damage following glial activation, depends on LFA-1/ICAM-1 interactions at the blood-brain barrier [123]. The expression level of endothelial ICAM-1 significantly influences the route of T cell transmigration: high ICAM-1 expression promotes rapid transcellular migration across the BBB, while moderate expression favors paracellular T cell trafficking [124]. Furthermore, during neuroinflammation, PECAM-1 in cerebral microvessels modulates the crawling phase of CD4^+^ T cells and regulates their infiltration *via* the transcellular pathway [125].

## Modes of T cell transmigration across the blood-brain barrier post-cerebral infarction

**Fig. 3:**
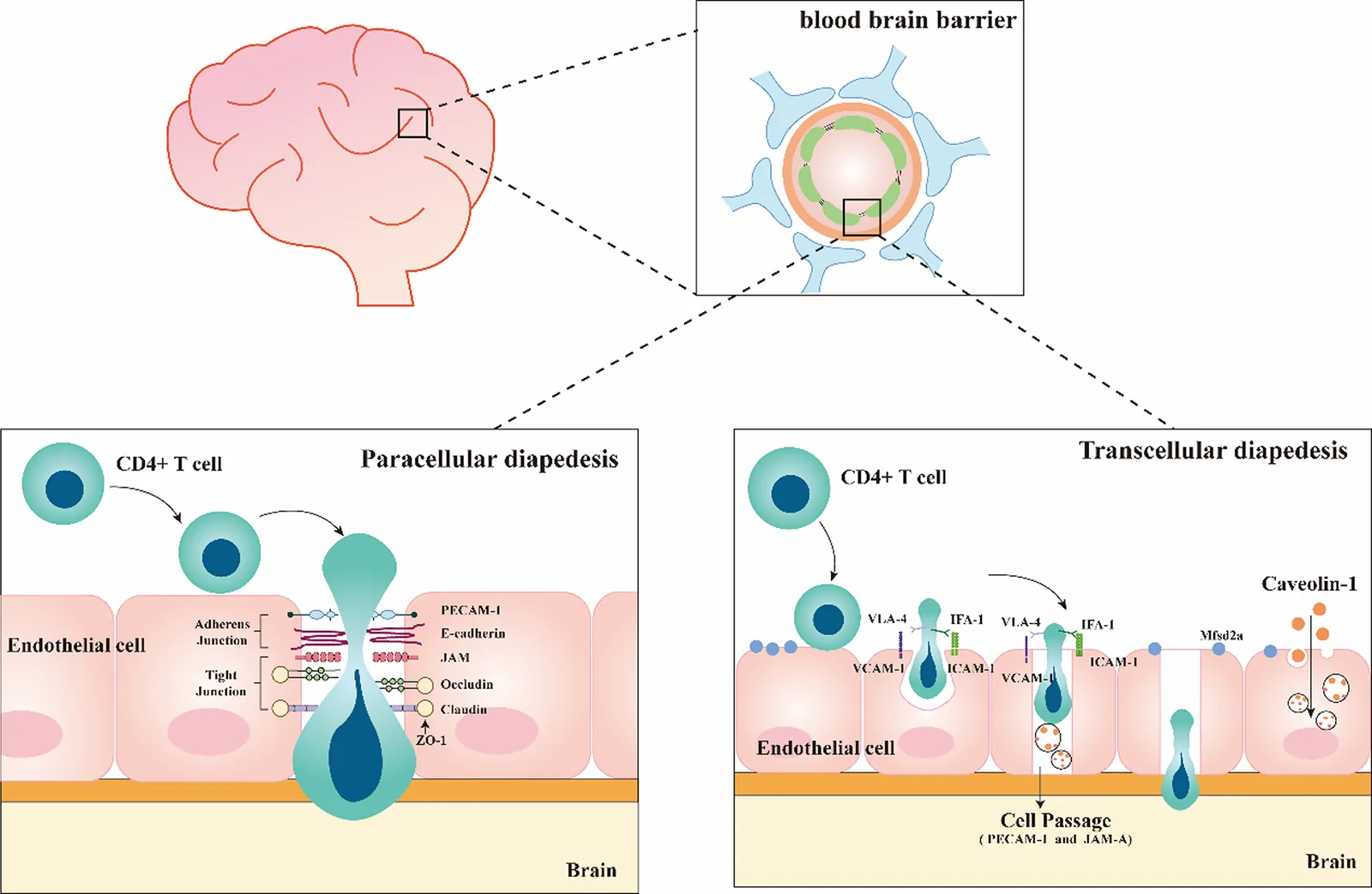
Two primary mechanisms for CD4^+^ T cell traversal across the blood-brain barrier. The penetration of CD4^+^ T cells across the blood-brain barrier occurs primarily through two distinct mechanisms: the paracellular pathway, which involves the disruption of tight junctions between endothelial cells, and the transcellular pathway, which is mediated *via* membrane pits through a process of transcytosis

Numerous studies have established that blood-brain barrier disruption represents an early pathological feature in several CNS disorders [126]. T cells primarily traverse this barrier through two distinct mechanisms: the paracellular pathway, mediated via endothelial junctional complexes between adjacent cells, and the transcellular pathway, facilitated by caveolae-dependent transcytosis across individual endothelial cells [98] (Fig. 3).

### Disruption of paracellular tight junctions

The BBB serves a critical protective function by excluding blood-borne substances from the CNS. This selective barrier function is primarily maintained by specialized intercellular junctions, particularly the tight junctions (TJs). TJs are macromolecular complexes that prevent paracellular diffusion of solutes across the cerebral endothelium. They work in concert with adherens junctions (AJs), another junctional complex located more basolaterally, to collectively establish the barrier’s tight seal. AJs are composed of cadherins that interact in the extracellular space, and catenins that link these cadherins to the actin cytoskeleton. The TJ complex comprises transmembrane proteins such as occludin, claudins, and junctional adhesion molecules (JAMs) that engage in heterophilic interactions across the intercellular space between adjacent endothelial cells. These membrane proteins are connected to the cytoskeleton through cytoplasmic adaptor proteins such as zonula occludens (ZO-1, -2, and − 3) and cingulin, which stabilize the junctional complex. Reduced expression or mislocalization of TJ components increases paracellular permeability, allowing solutes to traverse the BBB. This enhanced permeability facilitates the entry of inflammatory mediators including bradykinin, histamine, serotonin, arachidonic acid, and ATP into the brain parenchyma. These substances subsequently activate microglia, astrocytes, and pericytes, initiating a cascade of inflammatory pathways that further exacerbate BBB disruption and ultimately contribute to demyelination and neuronal injury.

### The caveolae-mediated transcellular pathway

As early as the 1970s, ultrastructural studies of the blood-brain barrier revealed that compromised cerebral endothelial cells can form patent vesicular channels, permitting the entry of large molecular tracers such as horseradish peroxidase into the CNS [127]. Similar vesicular structures have been identified in cerebral edema, traumatic brain injury, and sepsis [128]. Current research indicates that following cerebral ischemia, BBB endothelial cells employ caveolae for transcellular transport, positioning these structures and their associated proteins as a focus of scientific inquiry. Caveolae are formed by caveolin proteins, which induce invaginations of the plasma membrane to create characteristic Ω-shaped structures enriched with cholesterol and glycosphingolipids. As membrane-binding proteins, caveolins interact with cavin proteins in the cytoplasm to generate caveolar vesicles. Among the three caveolin isoforms, including Caveolin-1 (Cav-1), Caveolin-2 (Cav-2), and Caveolin-3 (Cav-3), brain microvascular endothelial cells (BMECs) predominantly express Cav-1 and Cav-2, while Cav-3 is primarily expressed in astrocytes. Crucially, Cav-1 is indispensable for caveola formation. Experimental evidence confirms that increased caveola density directly correlates with enhanced BBB permeability [129]. BBB integrity is further regulated by Major Facilitator Superfamily Domain Containing 2 A (Mfsd2a), which is upregulated during barrier maturation [130]. Mfsd2a specifically suppresses caveola-mediated transcytosis to maintain BBB function. Studies demonstrate that Mfsd2a is downregulated in mouse models of intracerebral hemorrhage, partially mediating BBB disruption [131]. Consequently, the formation of endothelial fenestrations via caveolar pathways may represent a significant mechanism of BBB leakage in certain pathological conditions. Notably, Mfsd2a expression begins selectively in vascular endothelial cells from embryonic day 15 in mice, where it inhibits endocytic activity in these cells, thereby restricting the paracellular passage of water-soluble substances into the brain. This evidence indicates that brain endothelial cells acquire a specialized lipid composition during embryonic development that effectively suppresses caveola-mediated transcytosis [132], highlighting the dynamic regulation of transcellular transport in both health and disease.

Early investigations into neuroinflammatory diseases revealed that lymphocytes predominantly traverse the blood-brain barrier *via* the transcellular pathway [133]. Subsequent research has demonstrated that T cells actually employ both paracellular and transcellular routes for CNS infiltration. In multiple sclerosis research, for instance, Th1 lymphocytes have been observed crossing the BBB through caveolae, while cell adhesion molecules including ICAM-1/2 and VCAM-1 accumulate within these structures, facilitating transcellular migration through interactions with enveloping endothelial membrane protrusions [134]. When CD4^+^ T cells adhere to the apical surface of inflamed brain endothelial cells, ICAM-1 and VCAM-1 cluster around the attached lymphocytes, promoting the formation of actin-rich caveolar structures that mediate their passage [134]. The actin-rich caveolar structures that mediate this transcellular passage are increasingly recognised as the product of a coordinated T cell–endothelial molecular dialogue. On the T cell side, adherent lymphocytes extend Src kinase– and WASP-dependent F-actin–rich protrusions whose LFA-1 (αLβ2) and VLA-4 (α4β1) integrins engage the apically clustered endothelial ICAM-1 and VCAM-1 [121]. On the endothelial side, this clustering, together with the additional recruitment of PECAM-1 and JAM-A, assembles a cup-like array of vertical microvillus projections that embraces the adherent T cell [135]. Concurrently, caveolin-1 enriched caveolae are recruited beneath the lymphocyte and progressively fuse to form the continuous transcellular channel through which the cell traverses the endothelium [136]. Interestingly, different T helper subsets appear to prefer distinct migration routes: tight junction remodeling primarily facilitates Th17 cell transmigration through the paracellular pathway, whereas caveolae-mediated transcytosis preferentially supports Th1 cell entry into the CNS [137]. Investigating these distinct T cell migration mechanisms may provide foundations for novel therapeutic strategies targeting specific neuroinflammatory pathways.

## Mechanistic pathways of CD4^+^ T cell transmigration across the BBB

### Wnt/β-catenin signaling pathway

**Fig. 4:**
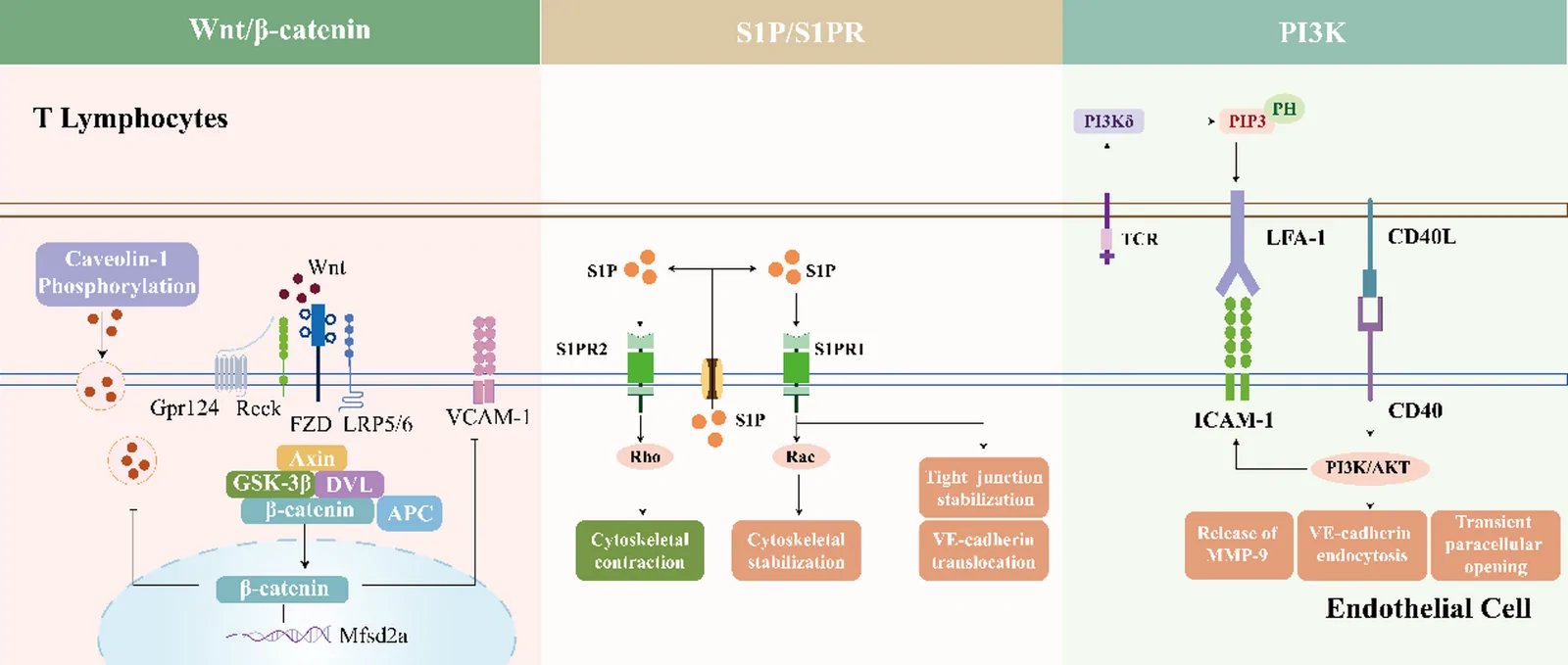
Mechanisms of CD4^+^ T cell traversal across the blood-brain barrier. Three signaling pathways mediate CD4^+^ T cell transmigration across the BBB. The Wnt/β-catenin pathway affects endothelial integrity by modulating tight junction proteins and endocytosis. The S1P/S1PR axis regulates T cell migration, inflammation and barrier function, partly through cytoskeletal rearrangement that alters permeability. The PI3K pathway acts on both sides of the BBB: in T cells, PI3Kδ potentiates LFA-1-mediated adhesion and migration, whereas at the endothelium, CD40L-CD40 engagement activates PI3K/AKT–p110α signaling that upregulates ICAM-1/VCAM-1, releases MMP-9 and opens VE-cadherin junctions to facilitate diapedesis

The Wnt signaling pathway has been increasingly recognized as a crucial regulator in maintaining cerebrovascular and neural cell function in recent years [138] (Fig. 4). Evidence indicates that endothelial Wnt/β-catenin signaling sustains adult BBB integrity by modulating both paracellular and transcellular pathways, while deficiencies in this pathway impair cerebrovascular and BBB development [139, 140]. In CNS endothelial cells, Wnt7a and Wnt7b activate β-catenin signaling through a ligand-specific co-receptor complex comprising GPR124 and RECK. GPR124, an orphan adhesion GPCR, binds RECK via its extracellular domain, enabling Wnt7-dependent recruitment of LRP5/6 to the GPR124–RECK heterodimer and assembly of a higher-order GPR124-RECK-Wnt7-LRP5/6 signaling complex. Notably, FZD is partially dispensable for Wnt7 signaling in brain endothelial cells: knockout of all FZD isoforms retains approximately 25% of Wnt7 responsiveness, whereas loss of GPR124, RECK, or LRP5/6 completely abolishes it [141]. Genetic ablation of Wnt7a/7b results in abnormal cerebrovascular development and impaired barriergenic functions [142]. Conversely, enhanced Wnt signaling activity significantly upregulates the expression of BBB-associated genes in cultured endothelial cells [143]. Endothelial-specific knockout of β-catenin reduces tight junction protein levels and disrupts intercellular junction ultrastructure. Furthermore, β-catenin deletion upregulates caveolin-1 expression, substantially increasing the number of endothelial caveolae and caveolae-mediated transcytosis [144]. Consistently, activation of the Wnt/β-catenin pathway correlates with increased tight junction protein expression and preserved BBB integrity following stroke [145]. The exceptionally low transcytosis rate represents another critical feature of the BBB. In the blood-retinal barrier, Wnt signaling directly regulates the transcription of the endothelial-specific transcytosis inhibitor Mfsd2a through a β-catenin independent mechanism. Mice lacking Lrp5 or the Wnt ligand Norrin exhibit increased retinal vascular leakage and enhanced endothelial transcytosis [146]. These findings suggest that the Wnt/β-catenin signaling pathway may influence CNS endothelial integrity by modulating transcytotic mechanisms [147]. Recent studies in cerebral ischemia-reperfusion animal models demonstrate significantly reduced Wnt/β-catenin signaling activity in ischemic brain tissue. Clinically, certain genetic variants of Lrp6 may be associated with IS risk [148]. Research in multiple sclerosis indicates that Wnt/β-catenin pathway inhibition during EAE/MS progression upregulates vascular cell adhesion molecule-1 and caveolin-1 expression, thereby promoting endothelial transcytosis, exacerbating clinical manifestations of EAE, and facilitating CD4^+^ T cell infiltration into the CNS. Reactivation of Wnt/β-catenin signaling in CNS vessels restores BBB integrity and limits immune cell CNS infiltration [134]. Accordingly, pretreatment with the β-catenin agonist SKL2001 reduces EAE severity and CD4^+^ T cell infiltration into the CNS [134].

### S1P/S1PR signaling pathway

Sphingosine-1-phosphate (S1P) is a membrane-derived signaling sphingolipid generated through ceramidase and sphingosine kinase (SphK1 and SphK2) activity. It primarily signals through S1P receptors (S1PRs) such as S1PR1 to regulate T cell migration, inflammatory responses, and vascular barrier function [149] (Fig. 4). Different cell types express distinct S1PR profiles that mediate specialized functions. Peripheral immune T cells express S1PR1, S1PR2, and S1PR4. These receptors detect S1P concentration gradients established by the SPNS2 transporter and activate downstream Gi protein signaling to direct T cell migration [150]. Research has revealed that S1P acts on both T cells and lymphatic endothelial cells in a coordinated manner: T cells respond to chemotactic signals through surface S1PR1 and S1PR4 receptors to regulate motility and adhesion, while lymphatic endothelial cells modulate intercellular junctions, adhesion molecules, and cytoskeletal organization through S1PR2, actively creating transmigration channels for T cells. This cooperative mechanism ultimately guides T cells to utilize the transcellular migration pathway [151]. Within the central nervous system, S1P activates several receptor subtypes expressed on endothelial cells and astrocytes that play crucial roles in regulating BBB integrity [149, 152]. Among the five S1P receptors (S1PR1-5), endothelial cells express S1PR1, S1PR2, and S1PR3, with S1PR1 and S1PR2 primarily mediating S1P-induced BBB functional changes [153]. S1P activation of S1PR1 strengthens cerebral endothelial tight junctions and adherens junctions, reduces BBB permeability, limits leukocyte infiltration, and inhibits astrocyte proliferation [152, 154]. S1PR1 enhances endothelial barrier properties by modulating the endothelial cytoskeleton [155]. The maintenance of tight junctions and adherens junctions, which function to exclude plasma solutes and restrict leukocyte infiltration into inflamed areas, requires cytoskeletal stability regulated by Rho family GTPases [156]. In vitro studies demonstrate that S1PR1 directly influences this pathway through Rho/Rac activation, leading to cytoskeletal rearrangement and cortical actin ring formation in endothelial cells [157]. Additional evidence suggests S1PR1 maintains BBB integrity during ischemic stroke by restricting vesicular transport, a key mechanism underlying acute-phase BBB dysfunction [158]. Studies confirm that S1PR1 activation effectively ameliorates ischemic stroke and protects the BBB [158]. S1P predominantly activates S1PR1 to enhance barrier function, while elevated plasma S1P levels activate S1PR2, inducing cytoskeletal rearrangement that disrupts BBB integrity [159]. S1PR2 plays a critical role in CNS inflammation by increasing BBB permeability and leukocyte entry [160–162]. Both in vivo and in vitro models indicate S1PR2 participates in endothelial inflammatory responses. Through RhoA/ROCK signaling activation, S1PR2 induces cytoskeletal contraction, stress fiber formation, and increased VE-cadherin phosphorylation, preventing its localization to cell-cell junctions and thereby enhancing barrier permeability and leukocyte entry [156, 163]. Moreover, the S1PR2 antagonist JTE-013 reduces expression of inflammatory molecules including E-selectin, VCAM-1, and CCL-2, consequently inhibiting immune cell transendothelial migration [152]. In AIS, SphK2-dependent S1P generation in brain tissue exerts neuroprotective effects, potentially through S1PR-mediated extracellular signaling. In contrast, S1PR2 induction in vascular endothelial cells following ischemia correlates with increased infarct size, edema, cerebrovascular permeability, hemorrhage, and neurovascular injury [164].

### PI3K signaling pathway

During T cell migration across the BBB, key steps including adhesion, directional migration, and transendothelial migration are critically regulated by the PI3K signaling pathway (Fig. 4). In T cells, PI3K, particularly the PI3Kδ (p110δ) isoform, is upregulated following activation of T cell receptors, co-stimulatory receptors, or cytokine receptors. This activation leads to the generation of phosphatidylinositol (3,4,5)-trisphosphate, which recruits pleckstrin homology domain-containing proteins to initiate downstream signaling. A key functional consequence of PI3K activation is the induction of a high-affinity conformation of LFA-1. This conformational enhancement promotes T cell adhesion to and migration across brain microvascular endothelial cells. Additionally, PI3Kδ signaling suppresses expression of CD62L and CCR7, thereby reducing T cell retention within lymph nodes and promoting their recruitment to inflammatory sites. This mechanistic shift enhances T cell migration potential toward cerebral inflammatory or injured regions [165].

On the endothelial side, PI3K signaling at the BBB is engaged through a direct CD4^+^ T cell–endothelial dialogue centred on the CD40L/CD40 dyad. Activated CD4^+^ T cells upregulate CD40L [166], which engages CD40, constitutively expressed on cerebral microvascular endothelial cells and further induced by TNF-α and IFN-γ during cerebral ischemia [167]. CD40 ligation recruits TRAF6 and c-Cbl to the p85 subunit of class IA PI3K, rapidly activating the endothelial PI3K/AKT axis [168]. This drives three pro-migratory events: upregulation of E-selectin, ICAM-1 and VCAM-1, supporting prolonged T cell capture and arrest [166]; release of MMP-9 to degrade the perivascular basement membrane [166]; and p110α-mediated Rac1 activation with Pyk2-dependent VE-cadherin phosphorylation [169], which disengages VE-PTP and p120-catenin to trigger clathrin-mediated VE-cadherin endocytosis and transient paracellular opening essential for diapedesis [170]. Together, the CD40L/CD40–PI3K/AKT–p110α axis is an endothelial-intrinsic mechanism by which activated CD4^+^ T cells orchestrate their own transmigration across the BBB.

## Molecular mechanism associated with CD4^+^ T cell subtype trafficking across the blood-brain barrier

### Molecular mechanisms involved in Th1 cells

#### Caveolin-1/CXCL10 signaling pathway

**Fig. 5:**
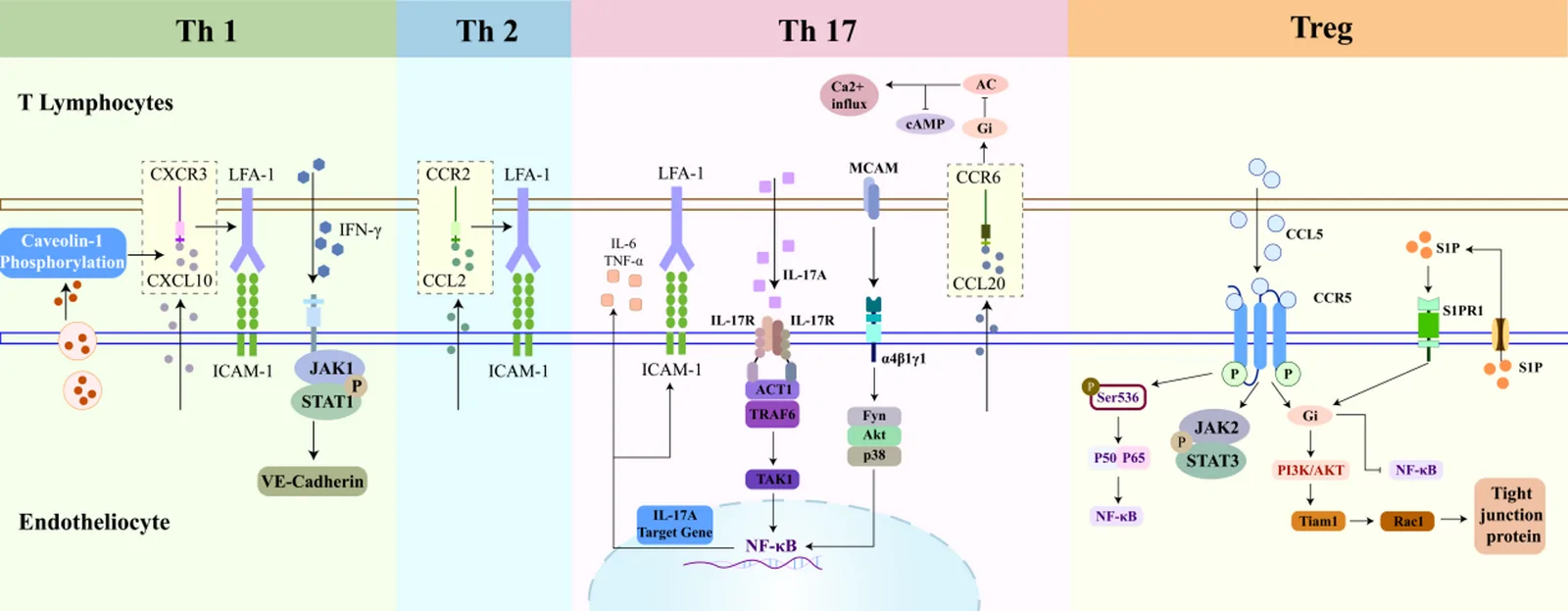
Mechanism Pathways of Major CD4^+^ T Cell Subtypes in Blood-Brain Barrier Infiltration. Specific molecular pathways dictate how different CD4^+^ T helper subsets traverse the blood-brain barrier. Th1 cell infiltration involves signals like Caveolin-1/CXCL10 and IFN-γ/IFNGR/JAK/STAT. Th2 cells rely on the CCR2-CCL2 axis and ICAM-1 for enhanced migration efficiency. In contrast, Th17 cells promote their own infiltration and amplify inflammation through mechanisms such as IL-17R/TRAF6/NF-κB, MCAM/Laminin-411, and CCL20/CCR6. Meanwhile, Treg cell migration to ischemic sites via CCR5/CCL5 creates a permissive environment for barrier infiltration

A recent study has identified the Caveolin-1/CXCL10 signaling pathway as a key mediator inducing CD4^+^ T cell migration across the blood-brain barrier via the transcellular route (Fig. 5). Specifically, CXCL10, a CXC chemokine, is produced and secreted into the brain parenchyma by CNS-resident cells upon stimulation by inflammatory signals such as IFN-γ [171]. Inflammatory cytokines enhance endothelial CXCL10 release and BBB disruption, ultimately promoting the infiltration of peripheral T lymphocytes [171]. CXCR3, the specific receptor for CXCL10, is highly expressed on activated T cells, particularly Th1 cells [172]. CXCL10-induced transcellular migration requires the expression of caveolin-1 and ICAM-1 in brain endothelial cells, as well as CXCR3 and LFA-1 on T cells. Caveolin-1 facilitates the clustering of CXCL10 into cytoplasmic reservoirs within brain endothelial cells, providing a mechanism to activate and recruit CXCR3^+^ T cells to cytoplasmic sites rather than to intercellular junctions for transmigration [173]. Thus, the neuroinflammatory chemokine CXCL10 promotes CD4^+^ T cell traversal across brain endothelium *via* the transcellular pathway, with endothelial caveolin-1 being essential for this CXCL10-induced migration [174]. Rapid binding of CXCR3 to its ligand CXCL10 enhances the adhesion of T cell integrin LFA-1 to endothelial ICAM-1 and induces transendothelial migration in vitro [173]. Furthermore, studies indicate that Th1 cells preferentially cross the BBB *via* caveolae, where adhesion molecules such as ICAM-1/2 and VCAM-1 become enriched and support transcellular migration through interactions with enveloping endothelial protrusions [124]. Activation of Cav-1 increases BBB permeability and is associated with α4 integrin/VCAM-1-dependent infiltration of Th1 lymphocytes into the CNS [175]. Notably, α4β1-integrin/VCAM-1 interactions mediate the arrest of encephalitogenic Th1 cells on spinal microvessels, whereas LFA-1/ICAM-1/2 interactions regulate Th17 cell adhesion to cerebral endothelial barriers. This suggests a preferential usage of α4-integrin by Th1 cells, while Th17 cells rely more heavily on αLβ2/ICAM-1 interactions [176].

#### IFN-γ/IFNGR/ JAK/ STAT signaling pathway

In immunology, IFN-γ serves as a pivotal cytokine driving Th1-type immune responses. It exerts its biological effects by binding to a specific cell surface receptor complex composed of IFN-γ receptor 1 and 2, thereby activating multiple signaling cascades across various cell types. Among these, the JAK–STAT pathway is the most extensively characterized [31] (Fig. 5). Upon IFN-γ stimulation, JAK1 and JAK2 mediate the phosphorylation and nuclear translocation of STAT1, leading to the expression of numerous interferon-stimulated genes that regulate immune cell activation, differentiation, and cytokine production [177]. While this pathway plays a central role in maintaining immune homeostasis and amplifying responses, its dysregulation is closely associated with the pathogenesis of various inflammatory and autoimmune disorders. Research indicates that the JAK-STAT pathway significantly influences BBB function and neuroinflammatory regulation. In vascular diseases and HIV-1-associated neuroinflammation, IFN-γ/STAT1 signaling activation suppresses the expression of tight junction proteins (e.g., claudin-5, ZO-1) in brain microvascular endothelial cells and upregulates adhesion molecules ICAM-1 and VCAM-1. These changes promote CD4⁺ T cell transendothelial migration and leukocyte infiltration, ultimately increasing BBB permeability [62, 178]. Some researchers propose that aberrant JAK-STAT signaling may represent a key molecular mechanism underlying inflammatory responses and barrier impairment in cerebrovascular diseases, suggesting that targeting this pathway holds potential for neuroprotective and anti-inflammatory therapies [179]. Beyond the CNS, IFN-γ signaling participates broadly in vascular endothelial regulation in other tissues. A recent study revealed that IFN-γ enhances glycolysis and lactate production in vascular endothelial cells, promoting VE-cadherin internalization and degradation, which increases vascular leakage [180]. Local blockade of IFN-γ signaling effectively restores vascular barrier function. Furthermore, comprehensive reviews indicate that IFN-γ coordinately regulates endothelial cytoskeleton and junctional integrity through multiple signaling axes, such as STAT1, p38 MAPK, small GTPases, and nitric oxide, thereby influencing barrier homeostasis [31].

### Molecular mechanisms involved in Th2 cells

In contrast to the extensively studied ability of Th1 and Th17 cells to cross the blood-brain barrier, the migration of Th2 lymphocytes into the CNS under neuroinflammatory conditions remains poorly understood. Studies indicate that compared to Th1 lymphocytes, both murine and human Th2 cells exhibit reduced adhesion to inflamed cerebral vessels, attributed to their lower expression of specific glycosylated epitopes on PSGL-1 [181]. Research utilizing in vitro models of the human BBB and blood-cerebrospinal fluid barrier has demonstrated that Th2 lymphocytes possess the lowest migratory capacity among T helper subsets [125, 182]. However, conflicting evidence from other in vitro experiments suggests Th2 cells can traverse endothelial layers more efficiently than Th1 cells, a process dependent on the CCR2-CCL2 axis and ICAM-1 [183] (Fig. 5). Recent findings confirm the critical role of ICAM-1 and, using human BBB models, indicate that CD99 contributes to Th2 cell migration, suggesting this mechanism may be relevant in human neuroinflammatory diseases [182]. Overall, the mechanisms governing Th2 cell transmigration across the BBB remain inadequately characterized, necessitating further investigation to elucidate the specific processes and molecular pathways involved.

### Molecular mechanisms involved in Th17 cells

#### IL-17R/ TRAF6 / NF-κB signaling pathway

Th17 cells exhibit a distinctive migration capacity, enabling efficient brain infiltration even during early inflammatory stages. These cells secrete IL-17, which induces endothelial injury and disrupts BBB integrity, thereby facilitating CNS infiltration by Th17 cells and other immune populations. Following stroke, infiltrated Th17 cells release IL-17 A, which persistently damages cerebral endothelial cells and exacerbates neuroinflammation through activation of the IL-17R/TRAF6/NF-κB signaling pathway [184, 185] (Fig. 5). Mechanistically, IL-17 A binding to its receptor on cerebral endothelial cells triggers recruitment of the ubiquitin ligase Act1 via the receptor’s intracellular SEFIR domain [186]. Act1 subsequently recruits TRAF6, leading to NF-κB activation [187]. This signaling cascade prolongs the mRNA half-lives of pro-inflammatory factors including TNF-α, IL-6, and ICAM-1, thereby amplifying endothelial inflammatory responses and exacerbating BBB disruption [188].

#### MCAM/ Laminin-411 signaling pathway

MCAM functions as an adhesion molecule that regulates CD4^+^ T cell migration and participates in CNS autoimmunity [189]. Among lymphocyte populations, MCAM expression is specifically associated with Th17 cells [189]. Laminin-411, a laminin isoform expressed within the vascular endothelial basement membrane under inflammatory conditions, serves as a ligand for MCAM [190]. The interaction between MCAM and laminin-411 facilitates Th17 cell tissue entry and promotes inflammatory responses [190] (Fig. 5). On the surface of pathogenic Th17 cells, highly expressed MCAM acts as a specific anchor, directly recognizing and binding with high affinity to laminin-411 within the basement membrane. This binding not only enables firm T cell adhesion to the basement membrane but also triggers intracellular signaling that promotes the secretion of matrix metalloproteinases. These enzymes locally degrade and remodel the extracellular matrix, creating a passage for T cells to efficiently traverse the basement membrane and infiltrate brain tissue, thereby driving inflammation [191].

#### CCL20/CCR6

At inflammatory sites within the brain, such as multiple sclerosis lesions, the chemokine CCL20 is produced. Th17 cells detect the CCL20 concentration gradient through their surface receptor CCR6 and are precisely recruited along this chemotactic gradient to the site of inflammation [192]. CCL20 plays a critical role in guiding Th17 cell migration to inflammatory loci and additionally contributes to IL-17 production [193]. Notably, CCR6-expressing Th17 cells demonstrate a hundredfold greater capacity for IL-17 production compared to their counterparts, establishing CCR6 as a key marker of pathogenic Th17 cells [192] (Fig. 5).

### Molecular mechanisms involved in Treg cells

The recruitment of Tregs involves sophisticated molecular interactions essential for their adhesion and targeted migration to specific regions. Tregs initially establish contact and undergo arrest primarily through LFA-1 binding to ICAM-1, subsequently initiating their transmigration process. During the acute phase of stroke (days 5–7 post-ischemia), Treg entry into the brain may occur through multiple coordinated mechanisms. First, following transient MCAO, Tregs rapidly upregulate CCR5 expression, while endothelial cells increase CCL5 production. This chemokine-receptor interaction facilitates Treg-endothelial engagement and guides Treg migration toward inflammatory sites [88]. Second, the establishment of a S1P gradient serves as another crucial guidance mechanism for Treg trafficking. S1P forms a concentration gradient that directs Treg movement from areas of lower concentration toward regions of higher S1P levels, thereby enhancing their localization to inflamed or damaged tissues [194]. Specifically, at 24 h post-stroke, measurements reveal the lowest S1P concentrations in the spleen, intermediate levels in circulation, and the highest concentrations within the ischemic core, creating a pronounced spatial gradient. This S1P distribution likely guides Treg migration from lymphoid reservoirs such as the spleen through peripheral circulation, ultimately promoting their accumulation in the ischemic brain region [195] (Fig. 5). Collectively, these complementary mechanisms enable Tregs to efficiently navigate to damaged cerebral areas, where they subsequently execute their immunomodulatory functions during stroke-induced inflammation.

## Therapeutic agents targeting T cells and their infiltration pathways (Table 1)

### Fingolimod

In 2010, fingolimod received FDA approval for the treatment of relapsing-remitting multiple sclerosis. Its primary mechanism involves acting as a selective sphingosine-1-phosphate receptor 1 antagonist, mediating irreversible receptor internalization in lymphocytes and sequestering T cells within lymph nodes and peripheral lymphoid organs [48]. Additionally, fingolimod downregulates endothelial expression of intercellular adhesion molecule-1, thereby reducing leukocyte adhesion to the vascular wall [196]. Other documented effects on the CNS include reduced neuronal apoptosis, protection of blood-brain barrier integrity, and modulation of local cytokine levels [197]. Administration of fingolimod immediately after reperfusion was shown to reduce infarct volume on days 1 and 3 in a murine model of transient cerebral ischemia [198]. This protective effect correlated with lymphocytopenia persisting for at least 7 days in both peripheral circulation and the cerebrovascular system, accompanied by reduced T cell infiltration into the brain parenchyma. In an open-label, evaluator-blinded, parallel-group trial (NCT02002390), 22 patients with cerebral infarction who were ineligible for alteplase treatment received daily oral fingolimod (0.5 mg) for the first 3 days post-stroke. The treatment demonstrated favorable tolerability and, compared to the standard treatment group, induced sustained lymphocytopenia for at least 7 days, resulting in reduced infarct volume at day 7 and improved neurological function at 3-month follow-up [199]. Furthermore, combination therapy with fingolimod and alteplase demonstrated enhanced protective effects compared to alteplase alone within both standard (4.5 h) and extended (4.5–6 h) time windows. These benefits included attenuated lesion growth, reduced hemorrhagic transformation, and improved 90-day neurological outcomes in 47 enrolled patients [200, 201].

### Adhesion factor antagonist (natalizumab and enlimomab)

Natalizumab is a humanized monoclonal antibody that specifically targets the α4 subunit of the α4β1 integrin adhesion molecule expressed on lymphocytes and neutrophils. By binding to this subunit, natalizumab effectively inhibits integrin-mediated interactions between these immune cells and endothelial cells, thereby preventing their infiltration into the brain parenchyma. Approved by the FDA in 2004 for relapsing-remitting multiple sclerosis, natalizumab has demonstrated therapeutic potential in experimental stroke models. Studies in animal models of cerebral infarction have shown that natalizumab treatment reduces the infiltration of both lymphocytes and neutrophils into the ischemic brain [202, 203]. A preclinical randomized controlled multicenter trial further revealed that the efficacy of natalizumab is model-dependent: it significantly reduced infarct volume in permanent middle cerebral artery occlusion models, but no comparable protective effect was observed in transient MCAO models [204]. A clinical study evaluated the effects of two doses of natalizumab on the functional prognosis of patients with acute ischemic stroke (AIS). The results showed that administering natalizumab within 24 h after the onset of acute ischemic stroke did not improve the prognosis of the patients [205]. Enlimomab, a murine-derived monoclonal antibody that targets CD54, was developed to test this approach. Although enlimomab reduced the infiltration of peripheral immune cells and the cerebral infarct volume in animal models, it worsened IS outcomes in clinical trials [206].

### Tacrolimus (FK506) and cyclosporin A (CsA)

The immunosuppressive mechanisms of both FK506 and CsA involve inhibition of calcineurin, leading to inactivation of nuclear factor of activated T-cells signaling pathways. Sharkey and Butcher initially demonstrated that FK506 administration at 60 min after occlusion exerts neuroprotective effects in an in vivo model of focal cerebral ischemia [207]. Studies have shown that treating animal models of acute ischemic reperfusion injury with LNP (FK-LNP) encapsulated with the brain-protective agent FK506 can effectively alleviate brain damage [208]. The cyclic undecapeptide cyclosporin A (CsA) is an effective immunosuppressant whose major mechanisms encompass inhibition of the immune system, particularly suppression of cytokine gene expression and inhibition of T lymphocytes. The drug blocks a calcium-sensitive T cell signal transduction pathway that regulates the transcription of genes expressed early during T cell activation [209]. The results of animal experiments showed that cyclosporin A could improve the levels of endothelin-1 and the infarct area in patients with transient cerebral ischemia [210], and also enhance the blood-brain barrier [211]. However, in clinical studies, Cyclosporine was generally not effective in reducing infarct size, although a smaller infarct size was observed in patients with proximal cerebral artery occlusion and efficient recanalization [212].

### Adhesion molecule and chemokine receptor antagonists

Chemokine receptors represent potential therapeutic targets for modulating T cell-mediated damage following stroke. These receptors are expressed on leukocytes and, through highly specific binding to chemokines released from damaged tissues, facilitate leukocyte migration to injury sites [213]. After reperfusion injury in mice, the upregulation of several chemokines in the brain enhances the recruitment of different leukocyte subsets [214]. Inhibiting chemokine/receptor interactions with small-molecule antagonists such as SB225002, a specific CXCR2 antagonist, can modify the leukocyte profile within the post-stroke brain [214]. Since these chemokine/receptor interactions contribute to the recruitment of detrimental T cell subsets and other leukocytes responsible for cerebral damage after stroke, targeting T lymphocyte migration to the brain rather than their activation may offer distinct therapeutic advantages. After a stroke, the expression of chemokines such as CXCL10 in the brain significantly increases. It binds to the CXCR3 receptor on the surface of T cells, guiding T cells to migrate towards the lesion site [172]. In intracerebral hemorrhage (ICH), a subtype of stroke, systemic administration of AMG487, a CXCR3 antagonist, plays a role in reducing the inflammatory response of the nervous system in cases of cerebral hemorrhage [215]. Two structurally distinct classes of CXCR3 antagonists have advanced into preclinical validation, piperazine-based piperidine compounds such as SCH 546,738, and 8-azaquinazoline derivatives such as AMG487, though neither has yet achieved clinical approval for neurological indications [216]. Chemokine receptor 5 (CCR5) has been found to be a potential therapeutic target for stroke [217]. A14 is a promising novel CCR5 antagonist for promoting neuronal repair after ischemic stroke. A14 treatment remarkably inhibited the reactive proliferation of glial cells after stroke and reduced the infiltration of peripheral immune cells [217]. Notably, CCR5 exhibits a context-dependent dual role: blockade on effector T cells reduces neuroinflammation, while CCR5 signaling on regulatory T cells facilitates their protective early recruitment to the ischemic endothelium, underscoring the importance of temporally defined therapeutic strategies [218].

### Traditional chinese medicine preparation

Herbal formulations demonstrate considerable multi-target therapeutic potential in regulating CD4⁺ T cell migration across the blood-brain barrier following ischemic stroke. Unlike single-target agents, TCM preparations are characterized by their capacity to simultaneously modulate multiple molecular pathways involved in BBB integrity, immune cell adhesion, and neuroinflammation, which may confer advantages in addressing the complex and dynamic pathophysiology of post-stroke immune injury. Huanglian Jiedu Decoction, a classic formula for clearing heat and toxins, inhibits CD4^+^ T cell infiltration across the BBB by suppressing LFA-1/ICAM-1-mediated cellular adhesion. Concurrently, it modulates the Wnt/β-catenin signaling pathway to improve both paracellular and transcellular routes of the BBB, thereby alleviating post-stroke neuroinflammatory injury [219]. Tongxinluo Capsule exerts protective effects through broad-spectrum inhibition of leukocyte-endothelial interactions, suppressing adhesion molecules including P-selectin, E-selectin, and ICAM-1, as well as chemokines CCL-2, CCL-3, CCL-4, CCL-5, and CXCL-1. In a murine transient MCAO model, intragastric administration of Tongxinluo significantly reduced infarction volume, alleviated BBB damage [220, 221].

Several other TCM-derived compounds have demonstrated the capacity to modulate T cell-mediated CNS injury following stroke. Baicalin, a flavonoid from *Scutellaria baicalensis*, reduces immune cell infiltration across the BBB by preserving tight junction integrity and promoting Foxp3⁺ regulatory T cell differentiation, thereby restraining effector T cell activity [222, 223]. Ginsenoside Rg1 blocks CKLF1-CCR5 protein interaction and restores CREB signaling to reduce peripheral immune cell CNS infiltration [224]. Collectively, these findings underscore the distinctive multi-target, multi-pathway therapeutic paradigm of TCM.

**Table 1:** Preclinical studies and clinical trials of T-cell targeted therapy

Treatment	Experimental model	Year	Sample Size	Drug Dose	Administration route	Molecular Targets	Outcomes	References
Fingolimod	Mouse transient MCAO	2011	Not mentioned	1 mg/kg once daily	Intraperitoneal	S1P receptor	Reduced infarct size, neurological deficit, edema and the number of dying cells in the core and periinfarct area	Wei et al. [189]
Fingolimod	Mouse transient MCAO	2013	235 C57BL/6 ( WT) and 24 Rag1−/− mice	1 mg/kg once daily	Intraperitoneal	S1P receptor	Reduced infarct size and improved functional outcome. Failed to prevent BBB breakdown and transendothelial immune cell trafficking	Kraft et al. [190]
Fingolimod	Clinical trial	2014	22 AIS patients	0.5 mg once daily	Orally	S1P receptor	Lower circulating lymphocyte counts, milder neurological deficits, better recovery of neurological functions, lesion size restrained, lower rT1%	Fu et al. [192]
Fingolimod	Clinical trial	2015	47 AIS patients	0.5 mg once daily	Orally	S1P receptor	Lower circulating lymphocytes, smaller lesion volumes, less hemorrhage, attenuated neurodeficits in National Institute of Health Stroke Scales, restrained lesion growth	Zhu et al. [193]
Fingolimod	Clinical trial	2018	46 AIS patients	0.5 mg once daily	Intravenous injection	S1P receptor	Better early clinical improvement, favorable shift of mRS distribution, greater reduction in perfusion lesion, suppressed infarct growth, improved anterograde reperfusion of downstream territory, prevented the failure of retrograde reperfusion from collateral circulation	Tian et al. [194]
Natalizumab	Mouse transient and permanent MCAO	2014	153 C57BL/6 mice	Administration of 300 µg was performed at two time points: 4 h prior to, or 3 h following the ischemia induction	Intraperitoneal	VLA-4	Reduced the invasion of T lymphocytes and neutrophils, failed to influence stroke outcome	Langhauser et al. [195]
Natalizumab	Mouse permanent MCAO	2015	120 C57BL/6 mice	Administration of 150 µg at the beginning of reperfusion and 24 h later	Intravenous injection	VLA-4	Reduced the infarct volume	Neumann et al. [196]
Natalizumab	Mouse transient and permanent MCAO	2015	315 C57BL/6 mice	Administration of 300 µg was performed at 3 h after stroke was induced	Intraperitoneal	VLA-4	Reduced both leukocyte invasion and infarct volume after the permanent MCAO, failed to reduce lesion size or leukocyte invasion after transient MCAO	Llovera et al. [197]
Natalizumab	Clinical trial	2020	277 AIS patients	single infusion at 2 different doses (300 and 600 mg)	Intravenous injection	VLA-4	No improvement was observed in the patient’s prognosis	Elkind et al. [198]
Enlimomab	Clinical trial	2001	625 AIS patients	160 mg on the first day, 40 mg for 4 days	Intravenous injection	ICAM-1	Worse mRS score, Fewer symptom-free recovery, more died	Enlimomab Acute Stroke Trial Investigators [199]
FK506	Rat MCAO	1994	Not mentioned	Administration of 0.03-1 mg/kg after endothelin injection	Intravenous injection	Calcineurin	Reduced volume of damage	Sharkey et al. [200]
LNP encapsulating FK506	Rat transient MCAO	2022	Not mentioned	100 µg/kg administered just after reperfusion	Intravenous injection	Calcineurin	Reduced damaged brain volume, ameliorated motor functional deficits	Yoneda et al. [201]
Cyclosporin A	Rat Endothelin-1-Induced tMCAO Model	2019	34 Sprague Dawley rats	CsA, 15 mg/kg	Intravenous injection	Calcineurin	CsA treatment thus ameliorated cerebral reperfusion metabolism and infarct size	Forsse et al. [203]
Cyclosporin	Clinical trial	2015	127 anterior-circulation stroke patients	cyclosporine, 2.0 mg/kg	Intravenous injection	Calcineurin	Cyclosporine was generally not effective in reducing infarct size. However, a smaller infarct size was observed in patients with proximal cerebral artery occlusion and efficient recanalization.	Nighoghossian et al. [205]
SB225002	Mouse MCAO	2011	54 C57BL/6 mice	1 mg/kg twice daily	Intraperitoneal	CXCR2	Reduced expression of CXC chemokine subfamily genes and neutrophil-related infiltration	Brait et al. [207]
AMG487, a CXCR3 antagonist	Collagenase-Induced ICH Model	2025	C57BL/6 mice	The working solution (30 mg/kg) was injected intraperitoneally 2 h after ICH induction, and then twice daily for 7 days.	Intraperitoneal	CXCR3	Systemic administration of AMG487, a CXCR3 antagonist, plays a role in reducing the inflammatory response of the nervous system in cases of cerebral hemorrhage	Ng et al. [208]
A14, CCR5 inhibitor	Focal Photothrombotic Ischemia Model	2023	C57BL/6J mice	A14, 20 mg/kg once daily beginning 24 h post stroke.	Intragastric administration	CCR5	A14 treatment remarkably inhibited the reactive proliferation of glial cells after stroke reduced the infiltration of peripheral immune cells and promoted neuronal repair after ischemic stroke.	Wu et al. [210]
Huang-Lian-Jie-Du decoction	Rat MCAO	2025	195 Sprague Dawley rats	0.45, 0.9, 1.8 g/kg/day	Intragastric administration	LFA-1/ICAM-1, Wnt/β-catenin	Improve neurological scores, reduce infarction volume, and mitigate pathological damage, reduce CD4^+^ T cell CNS infiltration	Chen et al. [212]
Tongxinluo	Mouse transient MCAO	2013	157 C57BL/6J male mice	TXL, 3.0 g/kg,	Intragastric administration	Adhesion molecules (P- selectin, E- selectin, and ICAM- 1) and chemokines CCL-2, CCL-3, CCL-4, CCL-5, and CXCL-1	Reduced infarction volume and BBB damage, Reduced leukocyte infiltration	Liu et al. [214]
Baicalin	In vitro T cell differentiation assay	2012	Not mentioned	0–40 µmol/L	In vitro	Foxp3; Treg differentiation	Dose-dependently promoted Foxp3⁺ regulatory T cell differentiation, thereby restraining effector T cell activity	Yang et al. [216]
Ginsenoside Rg1	Mouse MCAO	2024	Not mentioned	Not specified	Not specified	CKLF1/CCR5 axis; CREB signaling	Blocked CKLF1-CCR5 protein interaction, restored CREB signaling, reduced neuronal pyroptosis and peripheral immune cell CNS infiltration	Long et al. [217]

Notes: MCAO, Middle cerebral artery occlusion; BBB, blood-brain barrier; AIS, acute ischemic stroke; rT1%, relative T1% change; mRS, modified rankin scale; ICH, Intracerebral Hemorrhage

## Conclusion

Following AIS, the recruitment and infiltration of peripheral immune cells represent a critical mechanism underlying secondary brain injury, ultimately exacerbating blood-brain barrier disruption, promoting cerebral edema formation, and worsening neuroinflammation. This review systematically examines the dynamic changes of different CD4^+^ T cell subsets after stroke, their dual regulatory effects on BBB integrity, and the sophisticated multistep process of their transmigration across the BBB. This process involves sequential selectin-, integrin-, and ligand-dependent steps of capture, rolling, adhesion, and crawling, culminating in transmigration via either the paracellular or transcellular pathway. Notably, distinct CD4^+^ T cell subsets exhibit preferences for specific molecular toolkits and migratory routes. Th1 cells predominantly utilize the CXCL10/CXCR3 axis and transcellular migration, whereas Th17 cells rely more heavily on IL-17R/NF-κB signaling and MCAM/Laminin-411 interactions. Furthermore, the specific combinations of chemokines and their receptors expressed by different CD4^+^ T cell subsets provide spatiotemporal “address codes” for targeted trafficking, potentially contributing to secondary BBB disruption. Therapeutic strategies targeting CD4^+^ T cell infiltration have been extensively investigated in various conditions including autoimmune diseases, cancer, psoriasis, and multiple sclerosis, with numerous agents undergoing clinical trials. However, their application in ischemic stroke remains a significant and promising area for further exploration.

Current research has progressively elucidated the distinct temporal patterns and mechanistic routes by which different CD4^+^ T cell subsets infiltrate the blood-brain barrier. Although numerous immunosuppressive agents can inhibit T cell trafficking, acting primarily through Wnt/β-catenin signaling or chemokine-mediated regulation of ICAM or LFA-1, their clinical application faces limitations due to the complexity and plasticity of CD4^+^ T cell activation. Single-agent immunotherapy, despite its targeted specificity, may inadvertently trigger antagonistic effects among T cell subsets or promote compensatory pathways that ultimately drive disease progression. Consequently, post-stroke therapeutic strategies targeting CD4^+^ T cell infiltration should evolve from broad immunosuppression toward precise subset control. Future research should focus on identifying critical temporal windows for intervention, defining key subset transition nodes, and elucidating the corresponding signaling pathways to enable more precise immunomodulation. Although clinical trials of such approaches have yielded mixed outcomes, this remains a promising therapeutic direction. In summary, T cells play crucial roles in post-stroke inflammation and represent promising targets for immunotherapy in cerebral infarction.

## Future perspectives and therapeutic strategy considerations

Although our understanding of CD4^+^ T cell functions in ischemic stroke continues to advance, translating fundamental research into effective clinical therapies remains challenging. Current treatments targeting T cell migration, such as fingolimod and natalizumab, were primarily developed for chronic neuroinflammatory conditions like multiple sclerosis. Their application in AIS, which is a dynamic and rapidly evolving pathological process, requires more precise adaptation. Building on the systematic analysis of the complete CD4^+^ T cell infiltration process across the BBB and the associated subset-specific mechanisms delineated in this review, several promising directions for future therapeutic strategies emerge:

### Spatiotemporally precise interventions

The dominant CD4^+^ T cell subsets and their effects on the BBB vary significantly across different post-stroke phases. Consequently, therapeutic approaches should move beyond simple immunosuppression or enhancement toward temporal specificity. For instance, acute phase strategies might target Th1/Th17-specific migration mechanisms by blocking the CXCL10/CXCR3 axis, MCAM/Laminin-411 interactions, or IL-17 signaling, while subacute and recovery phases may require approaches that facilitate Treg homing and function.

### Targeting migration pathways rather than overall function

Complete T cell depletion or generalized immunosuppression carries significant risks of immunodeficiency. A more desirable strategy would specifically disrupt pathogenic trafficking without compromising systemic immune surveillance. This includes developing small-molecule inhibitors or monoclonal antibodies targeting adhesion molecule combinations preferentially expressed by specific subsets at the BBB, such as VLA-4/VCAM-1 for Th1 cells and LFA-1/ICAM-1/MCAM for Th17 cells.

### Multi-pathway coordinated intervention

Given that BBB disruption involves multiple signaling pathways, single-pathway targeting may yield limited efficacy. Developing combination therapies or bifunctional agents that simultaneously stabilize the BBB and inhibit specific lymphocyte migration pathways could produce synergistic protective effects. Traditional Chinese medicine, with its characteristic multi-target approach, may offer development potential in such coordinated interventions.

By deconstructing the multi-step, multi-subset process of T cell migration across the BBB with greater precision, we can potentially develop safer and more effective immunomodulatory strategies, thereby opening new therapeutic avenues for IS.

## Data Availability

No datasets were generated or analysed during the current study.

## Abbreviations

IS: Ischemic stroke
BBB: Blood-brain barrier
AIS: Acute ischemic stroke
CNS: Central nervous system
MCAO: Middle cerebral artery occlusion
APCs: Antigen-presenting cells
Th1: T helper 1 cell
Th2: T helper 2 cell
Th17: T helper 17 cell
Treg: Regulatory T cell
BMECs: Brain microvascular endothelial cells
PSGL-1: P-selectin glycoprotein ligand-1
LFA-1: Lymphocyte function-associated antigen 1
VLA-4: Very late antigen 4
ICAM-1: Intercellular Adhesion Molecule − 1
MCAM: Melanoma cell adhesion molecule
AJs: Adherens junctions
JAMs: Junctional adhesion molecules
Cav-1: Caveolin-1
Mfsd2a: Major Facilitator Superfamily Domain Containing 2A
